# Identification of Human Papilloma Viruses in Atheromatous Coronary Artery Disease

**DOI:** 10.3389/fcvm.2015.00017

**Published:** 2015-05-20

**Authors:** James S. Lawson, Wendy K. Glenn, Dinh D. Tran, Christopher C. Ngan, Johan A. Duflou, Noel J. Whitaker

**Affiliations:** ^1^School of Biotechnology and Biomolecular Sciences, University of New South Wales, Sydney, NSW, Australia; ^2^Department of Forensic Medicine, The University of Sydney, Sydney, NSW, Australia

**Keywords:** atheroma, coronary vessel anomalies, coronary artery disease, human papillomavirus, HPV E7 protein, inflammation

## Abstract

**Objective:**

To identify human papilloma viruses (HPV) in atheromatous coronary arteries.

**Background:**

Atheromatous arterial disease is primarily an initial inflammatory response to unknown stimuli. The crucial question is “what causes the initial inflammation in atheromatous disease?” HPV infections may be relevant as US women with vaginal, high risk for cancer, HPV infections, are at up to threefold increased risk of cardiovascular disease as compared with vaginal HPV-negative women. These studies did not include analyses of HPV in atheromatous coronary arteries.

**Methods:**

Atheromatous coronary arteries were identified and collected from 20 deceased donors. Polymerase Chain Reaction techniques were used to identify HPV gene sequences. Immunohistochemistry methods were used to identify HPV E7 proteins.

**Results:**

HPV types 16 and 18 were identified in 11 (55%) of 20 specimens. HPV E7 protein was identified in 10 (50%) of 20 specimens. Positive and negative HPV identification and HPV E7 expression in coronary smooth muscle cells were significantly correlated (cc = 0.503, *p* = 0.024). The HPV E7 proteins were expressed in smooth muscle cells and plasma cells, foam cells, and macrophages located in the atheromatous plaque. HPV E7 proteins were not expressed in infiltrating lymph cells.

**Conclusion:**

HPV gene sequences were identified in 55% of atheromatous coronary arteries and may have a role in coronary artery disease.

## Introduction

Atheromatous arterial disease begins as an inflammatory disease (1, 2). The crucial question is “what causes the initial inflammation?” Infectious microorganisms such as Epstein–Barr virus (EBV), herpes viruses, cytomegalovirus, chlamydia pneumonia, and porphyromonas gingivalis have all been postulated as having possible roles in atheromatous vascular disease (3).

Human papilloma virus (HPV) infections may be relevant as Kuo and Fujise have demonstrated that US women with vaginal, high risk for cancer, HPV infections are at approximately threefold increased risk of cardiovascular disease as compared with vaginal HPV-negative women (4). This study of 2,450 women was based on the US National Health and Nutrition Examination Survey, 2003–2006, and included HPV analyses of vaginal swab specimens and self-reported diagnoses of myocardial infarction and stroke.

However, Kuo and Fujise study did not include analyses of HPV in atheromatous coronary arteries. As we have not identified previous studies of HPV in coronary artery disease, we have conducted a preliminary study of HPV in coronary arteries from 20 deceased donors, all of whom died because of a myocardial infarction. We show that HPV gene sequences appear to be present in up to 55% of atheromatous coronary arteries.

## Materials and Methods

Archival formalin-fixed atheromatous coronary arteries were selected from 10 donors aged below 35 years, and 10 donors aged 35 years and over, from the Department of Forensic Medicine, Sydney, NSW, Australia. Younger and older age groups were selected as there may have been differences in the prevalence of HPVs identified in their coronary arteries. The cause of death for all 20 donors was a myocardial infarction due to atheromatous disease (as determined by Forensic Pathologists).

We sought to identify HPV by polymerase chain reaction techniques (PCR). It is important to note that HPV gene sequences in coronary artery material can be best identified by nested PCR, a method, which involves two sets of primers, used in two successive runs of the PCR.

Three HPV-positive and three HPV-negative specimens were selected from the 20 coronary artery specimens and were analyzed by PCR methods in an independent laboratory (QIMR Berghofer Medical Research Institute) using methods as per Antonsson et al. (5). PCR was repeated once on primary PCR amplicons in these confirmatory analyses. Different primers from those used at the University of New South Wales were used in these confirmatory analyses.

Human papilloma viruses E6 and E7 are part of HPV oncogenic mechanisms, which involve the degradation of p53 by HPV E6 protein and the inactivation of the p110^RB^ by HPV E7 protein. Positive outcomes of immunohistochemistry (IHC) analyses for these HPV proteins can offer supportive, but not conclusive, evidence with which to identify biologically active HPVs. Antibodies for the specific identification of HPV E7 proteins have recently been developed and made available on a commercial basis (6). This is the first use by IHC analyses using HPV E7 antibodies to identify these proteins in atheromatous cardiovascular disease. The HPV E7 antibodies (Cervimax) have been experimentally shown to be specific (6).

### Polymerase chain reaction for HPV

Genomic DNA was prepared using a Qiagen DNeasy blood and tissue kit, with the added step of incubating the paraffin sections at 120°C for 20 min in the ATL buffer before digestion with proteinase K. A reagent blank extraction control (an extraction without formalin fixed paraffin embedded – FFPE tissues) was performed during the DNA extraction procedure. The gDNA extracts were quantified with a NanoDrop spectrophotometer (Thermo Scientific). The DNA quality was tested by the amplification of a 268-bp fragment of the β-globin gene using primers G073 (5’-GAAGAGCCAAGGACAGGTAC-3’) and G074 (5’-CAACTTCATCCACGTTCACC-3’). The HPV genotypes were identified by BLAST via the US National Center for Biotechnology Information.

Round 1. My11 (5′GCACAGGGYCAYAAYAATGG3′) to modified Gp6 (5′AATCATATTCCTCTTCATGTC3′).
Round 2. Gp5 (5′TATTTGTTACTGTKGTWGATAC3′) to Gp6 (These primers were degenerated for HPV16 and 18, but are also capable of bringing up types 3, 11, 12, 45, 58, 73, and 75.)

Negative controls [no DNA (water) and a reagent blank] were used in parallel with all PCR analyses. Positive control (HPV18) was DNA extracted from HeLa cells.

Cycling conditions using Hot Start Taq Master Mix kit from QIAGEN: 95°C 15 min 1 cycle followed by 30 s 95°C, 30 s 55°C, 30 s, and 72°C 35 cycles.

Human papilloma virus identification was considered positive if sequences were identified at least twice in DNA extracted from the same specimens.

### Polymerase chain reaction for epstein–barr virus

Identification of EBV gene sequences by PCR was conducted on the same genomic DNA extracts as for HPV. The primers were as described in Glenn et al. (7).

### Immunohistochemistry

Standard manual IHC methods were used. The antibodies were HPV E7 monoclonal “Cervimax” – Valdospan GmbH. Austria (Catalogue VS 12001L).

The E7 antibody was optimized at 1/100 dilution. E7 proteins may be identified by Cervimax from a wide range of HPV types including HPVs 18, 39, 45, 59 of species 7, and HPV 16, 31, 33, 35, 52, 58 of species 9.

Human papilloma virus types 16 and 18 E7 proteins are detectable in both cell nuclei and cytoplasm. The outcomes were assessed by the intensity of staining on a scale of 0 and + to +++. Cervical cancer tissues were used as positive controls and normal breast tissues were used as negative controls. IHC with omission of the E7 antibody was negative.

### Statistical analysis

The correlations between the identification of HPV gene sequences and the expression of HPV E7 proteins were assessed with two-tailed Spearman’s non-parametric correlation statistic using the SPSS statistical package. A two-tailed *p* value of equal or less than 0.05 was considered to be statistically significant.

## Results

### HPV by PCR analyses

**Figure 1:**
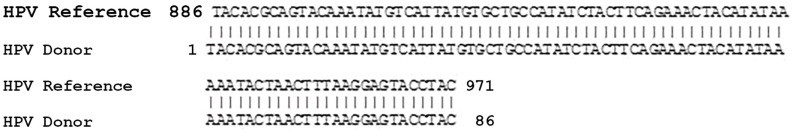
**HPV type 16 sequences identified in an atheromatous coronary artery from a 34-year-old male donor**. Reference sequence is from AF 393502.

As shown in Table 1, either or both HPV types 16 and 18 were identified in 11 (55%) of 20 specimens. Both HPV 16 and 18 are high risk for cancer HPV types. The positive control (HPV positive HeLa cells) was positive for HPV 16 and 18. The HPV negative outcomes for nine coronary artery donor specimens acted as negative controls for the PCR analyses. HPV type 16 sequences identified in an atheromatous coronary artery from a 34-year-old male are shown in Figure 1.

**Table 1:** **HPV sequences and HPV E7 protein expression in atheromatous coronary arteries from deceased donors selected by age**.

Donor	Age	HPV type	HPV E7 in smooth muscle cells
1	32	Neg	0
2	65	Neg	+
3	33	Neg	0
4	27	16/18	+
5	17	Pos	+
6	29	Neg	+
7	24	Neg	0
8	28	18	+
9	92	16/18	0
10	67	18	0
11	34	16/18	+++
12	33	Neg	0
13	76	16	0
14	71	Pos	+
15	49	18	+
16	43	Neg	0
17	59	18	+
18	69	Neg	0
19	35	16	+
20	74	Neg	0

*The correlation between positive and negative HPV gene sequence identification and HPV E7 protein expression in coronary smooth muscle cells is significant (cc = 0.503, *p* = 0.024 two-tailed Spearman non-parametric correlation). 16/18 means both HPV types 16 and 18 were identified*.

*Pos = positive, Neg = negative, HPV E7 by intensity of staining 0, +, ++, +++. There was insufficient material for HPV sequencing in two positive samples (the amplified PCR products were visualized on a 1.5% agarose gels)*.

**Figure 2:**
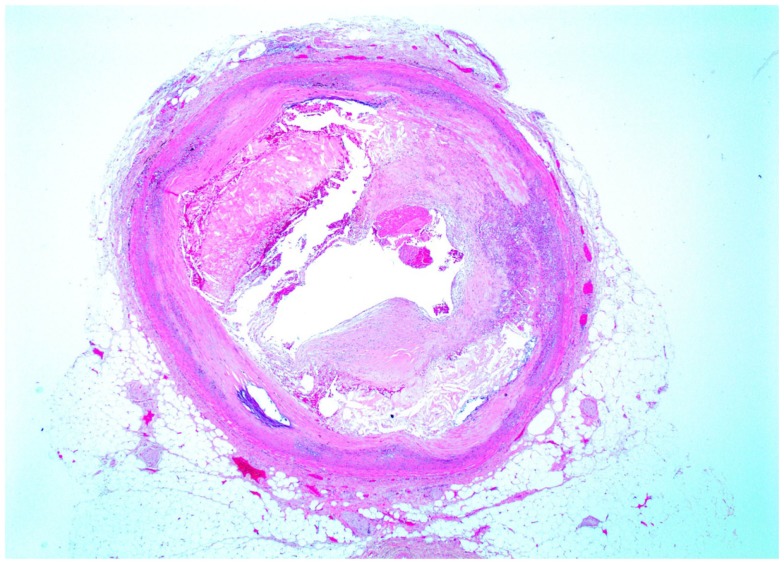
**Cross section of atheromatous coronary artery from a 35-year-old male donor**. This is the same specimen in which HPV E7 protein expression is shown in Figure 3.

A cross-sectional image of HPV type 16-positive atheromatous coronary artery disease is shown in Figure 2. This artery is from a 35-year-old male. The lumen of the coronary artery is almost completely blocked by atheroma.

**Figure 3:**
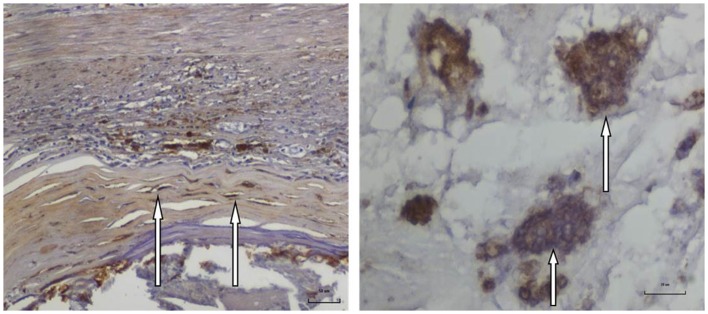
**HPV E7 protein expression in an atheromatous coronary artery specimen from a 35-year-old male donor**. Left: HPV E7 protein expression in atheromatous coronary smooth muscle cells. Right: HPV E7 protein expression in foam cells (foam cells are lipid laden macrophage cells associated with atheroma) in the same specimen.

Human papilloma virus type 18 was identified in two of the three HPV-positive coronary artery specimens (all three were HPV positive at the University of NSW laboratories) by the independent laboratory.

### EBV by PCR analyses

EBV gene sequences were identified in 6 (46%) of 13 coronary artery specimens (sound outcomes were not achieved in 7 specimens). This data is not shown in Table 1.

### HPV E7 protein

Human papilloma virus E7 was identified in 10 (50%) of 20 specimens as shown in Table 1. The correlation between positive and negative HPV gene sequence identification and HPV E7 protein expression in coronary smooth muscle cells is statistically significant (cc = 0.503, *p* = 0.024, two-tailed Spearman non-parametric correlation).

As shown in Figure 3, HPV E7 proteins were expressed in both atheromatous plaques and smooth muscle cells of atheromatous coronary arteries. The HPV E7 proteins were also expressed in plasma cells, foam cells, and macrophages located in the atheromatous plaque. HPV E7 proteins were not expressed in infiltrating lymph cells.

Positive HPV E7 protein expression was observed in smooth muscle cells in the walls of coronary arteries (not in the atheromatous plaque).

### Controls for IHC

**Figure 4:**
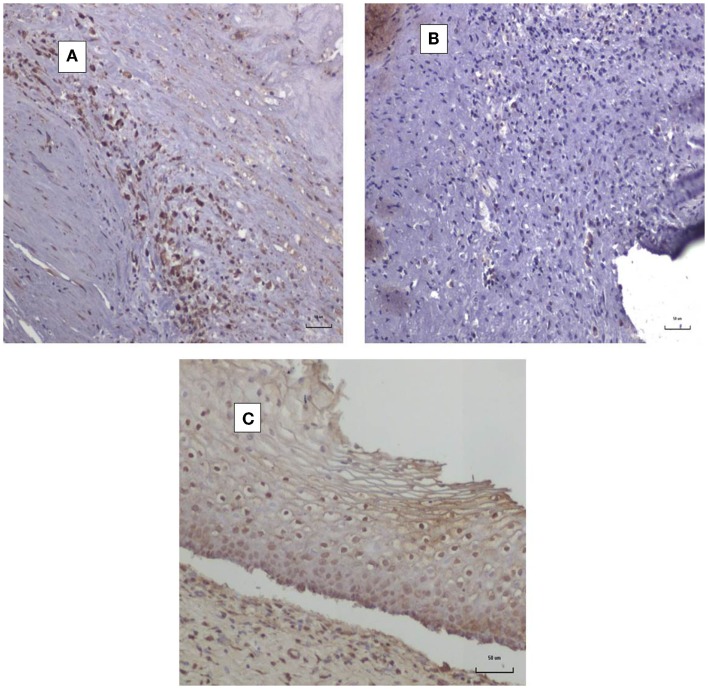
**HPV E7 protein expression in coronary atheroma**. **(A)** HPV E7-positive coronary atheromatous plaque; **(B)** HPV E7-negative coronary atheromatous plaque [different specimen to **(A)**]; **(C)** HPV E7-positive cervical intraepithelial neoplasia (CIN1).

It is difficult to obtain normal coronary arteries to act as negative controls for HPV E7 (Cervimax) antibodies. For this reason, we have shown images in Figures 4A,B of both positive and negative HPV E7 expression in atheromatous coronary arteries from two deceased donors using Cervimax. There were clearly negative outcomes using HPV E7 Cervimax antibodies in 10 of the 20 atheromatous coronary arteries used in this study. We regard these as suitable negative controls. As HPVs are involved in over 95% of cervical neoplasia, cervical specimens with cervical intraepithelial neoplasia provided readily available positive controls for HPV E7 Cervimax antibody. This is shown in Figure 4C. The staining is virtually identical with the same Cervimax antibody used on cervical CIN by Faoro et al. (8) who together with Pascale et al. (6) were the first to demonstrate the specificity of Cervimax antibodies.

## Discussion

In this study, both HPV types 16 and 18 gene sequences have been identified by PCR analyses in up to 55% of atheromatous coronary arteries. HPV E7 proteins were identified in 50% of the 20 coronary artery specimens. There was a consistent correlation between the identification of HPV gene sequences and the expression of HPV E7 proteins in coronary artery smooth muscle cells. However, the mere presence of HPV in coronary artery atheromatous plaques does not infer or conclude any mechanistic role of HPV in atherosclerosis.

Epstein–Barr virus sequences were identified in 46% coronary artery specimens (data not shown). This observation corroborates the previous identification of EBV in coronary artery disease (9).

An important limitation of this study is the lack of controls. This was due to the high prevalence of atheroma in coronary arteries of potential control donors who died in motor vehicle accidents.

Polymerase chain reaction amplification techniques are notoriously liable to contamination. However, false-positive PCR outcomes are unlikely as two HPV types were identified. If contamination had occurred, HPVs of the same types and sequences in a majority of specimens would have been observed. In addition, HPV type 18 was identified in the same specimens by an independent laboratory using different PCR methods and primers. This is an indication that the findings are valid. The positive identification of HPV E7 proteins also offers supportive, but not conclusive, evidence of the validity of the outcomes.

The correlation between the identification of HPV gene sequences and the expression of HPV E7 proteins in coronary artery smooth muscle cells is of particular interest. This correlation is specific to smooth muscle cells and not to other types of cells in atheromatous coronary arteries. It is of particular interest that positive HPV E7 staining was observed in smooth muscle cells within the walls of coronary arteries in addition to within atheromatous plaques. This suggests that HPVs may have influences within the wall structure of blood vessels. Bonin et al. (10) have shown that HPV E6 and E7 proteins induce proliferation of smooth muscle cells in aortic tissues. Given the correlation between HPV identification and HPV E7 protein expression in coronary artery smooth muscle cells, it is possible that HPV exerts a specific influence on these cells.

The concept that HPV and other infections may have an initiating role in atheromatous coronary artery disease is biologically plausible. This view is based on the following evidence: (i) initially atheromatous arterial disease is primarily an inflammatory phenomena (1), (ii) HPV infections commonly elicit a persistent inflammatory response (11, 12), (iii) HPV genes E6 and E7 can extend the life span and immortalize atheromatous plaque-derived normal human aortic smooth muscle cells (10), (iv) there is a positive association between HPV infections and giant cell arteritis of the temporal artery (13), (v) HPV type 16 has been identified in vascular endothelial cells adjacent to cervical cancer (14), and (vi) the E5 protein of HPV type 16 modulates composition and dynamics of membrane lipids in keratinocytes (15). Caspases are protein enzymes, which influence cell death (apoptosis), and have a critical role in vascular inflammation and development of atherosclerosis (16). Recently, it has been demonstrated that HPV E6 oncoproteins promote nuclear localization of active caspase 8 (17).

There are many factors, which contribute to atheromatous vascular disease including low density lipoproteins, cigarette smoking, hypertension, diabetes mellitus, and genetic susceptibility. The possibility that infections may have a role in atheromatous vascular disease is highlighted by the long-term pattern of deaths due to myocardial infarction. Deaths due to acute myocardial infarction among Western men and women rose dramatically (from 200 to 300 deaths per 100,000 people per year) between 1920 and 1950 and then fell equally dramatically to the 1980s (18). These death rates do not parallel dietary changes during the same period. This pattern of a rapid rise and fall in death rates is typical of infectious and not dietary influences.

Other viruses may alter lipid metabolism in coronary cells. For instance, atherosclerotic lesions are induced by infections with Marek’s disease herpes virus in chickens (19), herpes simplex virus infections of arterial smooth muscle cells can lead to marked accumulation of cholesterol (20), and herpes simplex virus-1 infections increase binding of low density lipoproteins to arterial smooth muscle cells (21).

It is commonly accepted that HPV infections are predominantly transmitted during sexual activities and that they are confined to epithelial cells. There is evidence that neither of the notions is wholly true. HPVs have been repeatedly identified in white blood cells in circulating blood of normal subjects and patients with HPV-associated cervical cancer (22). HPVs have been identified in mucosal cells and saliva of newborn infants and children (23). In addition to epithelial cells, HPVs have been identified in glioblastomas, neural structures, and vascular endothelial cells (20, 24). Accordingly, it is plausible that HPV s may spread by bloodstream transmission to vascular structures.

When considered together with these many prior observations, plus the epidemiological evidence that women with HPV genital infections are at up to threefold increased risk of heart attacks or stroke, the notion that HPV infections may have an active role in atheromatous vascular disease is of considerable interest. However, the data do not exclude the possibility that HPVs in atheroma and coronary artery smooth muscle cells are merely opportunistic parasites and not causal.

Our preliminary investigations need to be confirmed, extended, and evidence of HPV biological activity in coronary arteries needs to be developed.

## Author Contributions

All authors contributed substantially to the present work, revised the work critically, approved the final paper, and agreed to be accountable for all aspects of the work.

## Ethics Statement

This study was formally approved by the Human Research Ethics Committee of the Sydney Local Health District number 13/0048 and by the University of New South Wales Human Research Ethics Committee number HREC HC11421.

## Conflict of Interest Statement

The authors declare that the research was conducted in the absence of any commercial or financial relationships that could be construed as a potential conflict of interest.

## Abbreviations

EBV, Epstein–Barr virus; HPV, human papilloma virus; IHC, immunohistochemistry; PCR, polymerase chain reaction.
